# Accurate genetic diagnosis of Finnish pulmonary arterial hypertension patients using oligonucleotide-selective sequencing

**DOI:** 10.1002/mgg3.147

**Published:** 2015-04-25

**Authors:** Sanna Vattulainen, Joonas Aho, Pertteli Salmenperä, Siina Bruce, Jonna Tallila, Massimiliano Gentile, Marja Sankelo, Tarja Laitinen, Juha W Koskenvuo, Tero-Pekka Alastalo, Samuel Myllykangas

**Affiliations:** 1 Pediatric Cardiology, Children’s Hospital Helsinki, University of Helsinki and Helsinki University Central HospitalHelsinki, Finland; 2 Research Centre of Applied and Preventive Cardiovascular Medicine, University of TurkuTurku, Finland; 3 Blueprint GeneticsHelsinki, Finland; 4 Department of Internal Medicine, Tampere University HospitalTampere, Finland; 5 Department of Pulmonary Diseases and Allergology, Turku University Hospital and University of TurkuTurku, Finland; 6 Department of Clinical Physiology and Nuclear Medicine, HUS Medical Imaging Center, Helsinki University of Central Hospital and University of HelsinkiHelsinki, Finland; 7 Department of Biochemistry and Developmental Biology, Faculty of Medicine, University of HelsinkiHelsinki, Finland

**Keywords:** BMPR2, genetic diagnostics, genetics, OS-Seq, pulmonary arterial hypertension

## Abstract

The genetic basis of pulmonary arterial hypertension (PAH) among Finnish PAH patients is poorly understood. We adopted a novel-targeted next-generation sequencing (NGS) approach called Oligonucleotide-Selective Sequencing (OS-Seq) and developed a custom data analysis and interpretation pipeline to identify pathogenic base substitutions, insertions, and deletions in seven genes associated with PAH (*BMPR2*, *BMPR1B*, *ACVRL1*, *ENG*, *SMAD9*, *CAV1*, and *KCNK3*) from Finnish PAH patients. This study represents the first clinical study with OS-Seq technology on patients suffering from a rare genetic disorder. We analyzed DNA samples from 21 Finnish PAH patients, whose *BMPR2* and *ACVRL1* mutation status had been previously studied using Sanger sequencing. Our sequencing panel covered 100% of the targeted base pairs with >15× sequencing depth. Pathogenic base substitutions were identified in the *BMPR2* gene in 29% of the Finnish PAH cases. Two of the pathogenic variant-positive patients had been previously tested negative using Sanger sequencing. No clinically significant variants were identified in the six other PAH genes. Our study validates the use of targeted OS-Seq for genetic diagnostics of PAH and revealed pathogenic variants that had been previously missed using Sanger sequencing.

## Introduction

The role of genetic diagnostics in the evaluation of patients with Mendelian disorders is increasing rapidly in all fields of medicine. Understanding the underlying genetic causes of inherited diseases can contribute to the treatment and follow-up strategies, and may bring psychological comfort for the patient. Moreover, genetic diagnosis of the proband can allow effective risk assessment of family members, rationalize follow-up strategies, and enable earlier interventions.

Pulmonary arterial hypertension (PAH) is a severe and progressive disease characterized by vascular remodeling of small pulmonary arteries resulting in an increase in pulmonary arterial pressure, and eventually leading to right heart failure (Runo and Loyd 2003). Despite the improvement of treatments, PAH still remains a fatal disease with high mortality (Chakinala 2005). While the pathogenesis of idiopathic and hereditary forms of PAH (IPAH and HPAH) is a complex and incompletely understood, their genetic basis is well recognized. Hundreds of mutations in eight genes have been reported to associate with both IPAH and HPAH. HPAH is commonly regarded as an autosomal dominant disease (Larkin et al. 2012; Machado et al. 2009).

Mutations in the bone morphogenetic protein receptor 2 (*BMPR2*) gene, a member of the transforming growth factor beta (TGF-*β*) superfamily, are currently considered as main causes for the pathogenesis of PAH (Machado et al. 2006). Over 300 different *BMPR2* mutations have been identified in PAH patients (Machado et al. 2009, 2001). Mutations in the *BMPR2* gene have been identified in up to 75% of HPAH cases and in 25% of IPAH cases. Previously, the penetrance of PAH among *BMPR2* mutation carriers was considered low, however, a recent study based on the Varderbilt Pulmonary Hypertension Registry found an overall penetrance of 27%, (42% in females and 14% in males). The disease penetrance could be even higher as the disease manifestation may be delayed for 75 years and express phenotypic variability (Larkin et al. 2012; Machado et al. 2009; Soubrier et al. 2013).

Other genes than *BMPR2* have been implicated in rare cases of PAH. Mutations in bone morphogenetic protein receptor 1B (*BMPR1B*), activin-like kinase-type 1 (*ACVRL1*; *ALK1*), endoglin (*ENG*), smad family member 9 (*SMAD9*), caveolin 1 (*CAV1*), potassium channel subfamily K, member 3 (*KCNK3*), and *NOTCH3* have been described in the development of the disease (Austin et al. 2012; Chaouat et al. 2004; Chida et al. 2012a; Harrison et al. 2003; Li et al. 2009; Ma et al. 2013; Shintani et al. 2009). The role of these genes in the pathogenesis of IPAH and HPAH is suggested significantly smaller compared to *BMPR2*.

The genetic factors contributing to PAH in Finland, a genetic isolate in Europe, has not been comprehensively evaluated. In this study, we analyzed Finnish PAH patient isolate to detect pathogenic or likely pathogenic variants associated with PAH by utilizing the novel-targeted Oligonucleotide-Selective Sequencing (OS-Seq) technology (Myllykangas et al. 2011). We designed target-specific oligonucleotides to capture all coding exons, exon-intron boundaries, and known intronic mutations in the PAH-associated target genes. All these target genes were comprehensively analyzed using OS-Seq. These 21 Finnish PAH patients had been previously tested for mutations in the *BMPR2* and *ACVRL1* genes using Sanger sequencing (Sankelo et al. 2005). With OS-Seq we detected all previously identified pathogenic or likely pathogenic variants of *BMPR2* gene and identified two new pathogenic *BMPR2* variants that were previously missed using Sanger sequencing. Our results validate the use of OS-Seq panel for diagnostics of PAH.

## Methods

### Patient samples

Blood samples for genetic studies were obtained from Finnish patients diagnosed with PAH under the approval from the Ethical Committees of the five University Hospitals and by the Ministry of Social Affairs and Health of Finland. All participants provided the written informed consent for genetic analysis as previously reported (Sankelo et al. 2005). Genomic DNA was extracted from the blood samples (Sankelo et al. 2005) and DNA samples of 21 Finnish iPAH (*N* = 18) and HPAH (*N* = 3) patients were selected for the study.

### PAH panel

**Figure 1:**
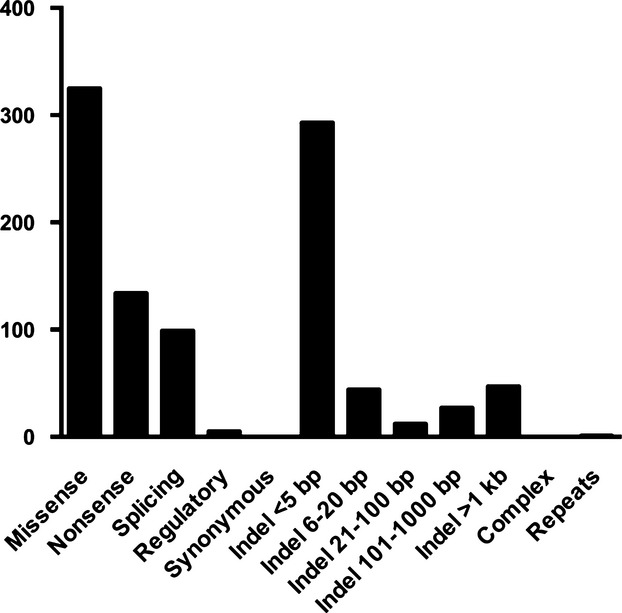
Distribution of mutations in genes selected for diagnostics of PAH. Mutations in seven PAH-associated genes were collected from the literature.

Seven genes were selected for the PAH panel based on literature search in 2012-2013 (*BMPR2*, *BMPR1B*, *ACVRL1*, *ENG*, *SMAD9*, *CAV1*, and exon 2 of *KCNK3*) and all validated information about the mutations were incorporated into the bioinformatics pipeline (Table1 and Fig.1). Noteworthy, exon 1 of *KCNK3* was omitted in the analysis because it is difficult to sequence due to extremely high GC content and because all mutations in *KCNK3* that have been linked with PAH are located in exon 2 (Ma et al. 2013).

**Table 1:** Genes selected for diagnostics of PAH

Gene symbol	Gene name	Reference
*BMPR2*	Bone morphogenic protein receptor 2	Machado et al. (2006)
*BMPR1B*	Bone morphogenic protein receptor 1B	Chida et al. (2012a, b)
*ACVRL1*	Activin A receptor type II like-1	Harrison et al. (2003)
*ENG*	Endoglin	Chaouat et al. (2004)
*SMAD9*	Smad family member 9	Shintani et al. (2009)
*CAV1*	Caveolin 1	Austin et al. (2012)
*KCNK3*	Potassium channel, subfamily K, member 3	Ma et al. (2013)

### Sequencing

The quality of genomic DNA samples was evaluated using gel electrophoresis and DNA concentration was measured using Qubit (Life Technologies, Grand Island, NY). Genomic DNA samples were fragmented using Fragmentase (New England Biolabs, Ipswitch, MA) and sequencing libraries were prepared using NEBNext Ultra (New England Biolabs) and PCR amplified. Target-specific oligonucleotides were designed to capture all coding exons, exon-intron boundaries, and known intronic mutations in the PAH-associated target genes and obtained from Integrated DNA Technologies (Leuven, Belgium). Exon definitions for target genes were derived from the CCDS. The targeting and sequencing of DNA was automated using the MiSeq Desktop Sequencer (Illumina, San Diego, CA). Pathogenic and likely pathogenic mutations found in the patient samples were confirmed using Sanger sequencing.

### NGS data analysis

Primary data analysis was carried out by Illumina’s MiSeq Reporter software using the generateFASTQ workflow, that generates a single FASTQ file with base calls and Phred quality scores for R1 and R2 reads, separately. The data were transferred to a dedicated pipeline server for downstream analysis, which started with extraction of data for individual samples using a proprietary demultiplexing algorithm that analyses the 9 bp long index adapter sequence of each read to determine sample identity. Sequence reads were then trimmed to exclude adapter and capture oligo sequences using a proprietary algorithm that estimates the size of captured target DNA based on the genomic coordinates of read mate-pairs. Low-quality data were identified and removed using the Prinseq package (Schmieder and Edwards 2011) to exclude reads with more than 10% of unclassified nucleotides, as well as trimming nucleotides with Phred scores inferior to 20 from starting from the 3′- end. The cleaned-up sequence reads were subsequently aligned to the human reference sequence assembly GRCh37 using the Burrows-Wheeler Aligner (Li and Durbin 2009) in single-end mode with default parameters. Variant calling was performed using the Genome Analysis Tool Kit (GATK) (McKenna et al. 2010) following the guidelines outlined in the document “Best Practice Variant Detection with the GATK (v1.x)” issued by the developers.

### Variant interpretation

An automated bioinformatics pipeline was applied to identify pathogenic base substitutions, insertions and deletions (INDELs) in PAH-associated genes. Mutations that have been shown to be associated with PAH were collected from original research publications and existing mutation databases and queried using patients’ variants. Population frequencies for detected variants were derived from the Exome Variant Server (NHLBI GO Exome Sequencing Project (ESP), Seattle, WA (http://evs.gs.washington.edu/EVS) and the Exome Aggregation Consortium (ExAC), Cambridge, MA (http://exac.broadinstitute.org). PolyPhen, SIFT (Adzhubei et al. 2010; Ng and Henikoff 2001, 2002), and Mutation Taster (Schwarz et al. 2014) tools were used to predict the effects of variants on the protein structure and functions. Variants were classified as pathogenic or likely pathogenic when previous studies had identified them in other PAH patients. Furthermore, pathogenic gene variants associated with PAH are considered to be extremely rare or absent in control populations and often considered deleterious by in silico prediction.

## Results

Here, we present the first clinical study on Finnish PAH patients using the novel-targeted OS-Seq technology. We confirmed pathogenic or likely pathogenic variants of *BMPR2* in four patients and detected two new pathogenic variants of *BMPR2* gene that were previously missed using Sanger sequencing (Sankelo et al. 2005). Four out of six patients were carrying pathogenic gene variants that were predicted to cause a truncated BMPR2 protein: the point mutations c.994C>T (p.Arg332X) in the kinase domain and c.1750C>T (p.Arg584X) in the C-terminal cytoplasmic domain, and a deletion causing a frameshift mutation (c.1376_1377delGA, p.Arg459ThrfsX11) in the kinase domain in two patients. In addition, two of the patients had missense point mutations in *BMPR2*: c.1472G>A (p.Arg491Gln) located in a kinase domain and c.2696G>C (p.Arg899Pro) located in C-terminal cytoplasmic domain (Table2). Both of these variants featuring missense mutations were classified as deleterious in all in silico variant effect prediction analyses. In addition to these five pathogenic variants, three other variants were detected in *BMPR2*. Polymorphisms were observed in two patients and one short GGC-insertion in the promoter was identified in three patients (Table3). The clinical significance of the identified GGC-insertion is unknown, since the frequency of this variant is unknown in control populations, as the region is not covered in these databases. Altogether, our OS-Seq panel revealed six (29%) PAH patients carrying a pathogenic, or likely pathogenic, variant of *BMPR2* gene (Table2).

**Table 2:** Mutations in *BMPR2* in Finnish iPAH and hPAH patients

Patient	1	2	3	4	5	6
Sex	F	F	M	M	F	F
Age of diagnosis	31	52	35	49	19	47
Family history	No	No	Yes	No	No	No
Exon number	8	10	11	12	10	12
Nucleotide change	c.994C>T	c.1376_1377delGA	c.1472G>A	c.2696G>C	c.1376_1377delGA	c.1750C>T
Amino acid change	p.Arg332X	p.Arg459ThrfsX11	p.Arg491Gln	p.Arg899Pro	p.Arg459ThrfsX11	p.Arg584X
Mutation type	Stop	Frameshift	Missense	Missense	Frameshift	Stop
Protein domain	Kinase domain	Kinase domain	Kinase domain	C-terminal cytoplasmic domain	Kinase domain	C-terminal cytoplasmic domain
Detected previously by Sanger Sequencing	Yes	No	Yes	Yes	Yes	No
Mutation in other genes	None	ACVRL1*	None	None	None	None

Likely benign (c.536A>C, p.Asp179Ala). Mutation nomenclature is based on GenBank accession NM_001204.6 with nucleotide on being the first nucleotide of the translation initiation codon.

* Refers to ^*^ Likely benign (c.536A<C, p.Asp179Ala).

**Table 3:** Variants identified in Finnish PAH patients

Gene	Variant ID	Consequence	Clinical significance	Variant prediction	MAF^1^	Number of patients
ACVRL1	CM033589	Missense	Likely benign	Tolerated	0	1
BMPR2	rs137852751	Stop	Pathogenic	Disease-causing	0	1
BMPR2	–	Frameshift	Pathogenic	Disease-causing	0	2
BMPR2	rs137852752	Missense	Pathogenic	Disease-causing	0.000008134	1
BMPR2	rs137852749	Missense	Pathogenic	Disease-causing	0	1
BMPR2	CM010166, COSM209591	Stop	Likely pathogenic	Disease-causing	0	1
BMPR2	rs1061157	Synonymous	Benign	Neutral	0.1201	7
BMPR2	rs375624016	5′UTR-insertion	Unknown	Unknown	0	3
BMPR2	rs55722784	Synonymous	Benign	Neutral	0.01371	2
ENG	rs3739817	Synonymous	Benign	Neutral	0.09142	4
ENG	rs34828244	Synonymous	Benign	Neutral	0.01042	2
ENG	rs36092484	Synonymous	Benign	Neutral	0.01801	2
ENG	rs16930129 (rs11545664)	Synonymous	Benign	Neutral	0.0983	4
ENG	rs35400405	Missense	Benign	Tolerated	0.04790	2
ENG	rs7847860	Intron	Benign	Neutral	0.06638	3

Minor allele frequencies were collected from the ExAC-project data.

In addition, seven variants were detected in *ACVRL1* and *ENG* (Table3). One patient carried a gene variant featuring a novel missense mutation c.536A>C (p.Asp179Ala) in a serine and threonine residue-rich region in the *ACVRL1* gene. This *ACVRL1* variant was predicted to be benign by in silico analyses. This patient also tested positive for a pathogenic variant featuring the frameshift mutation in *BMPR2* (Table3), and therefore the *ACVRL1* variant was classified as likely benign. The variants identified in the *ENG* gene were not considered deleterious (Table3).

**Figure 2:**
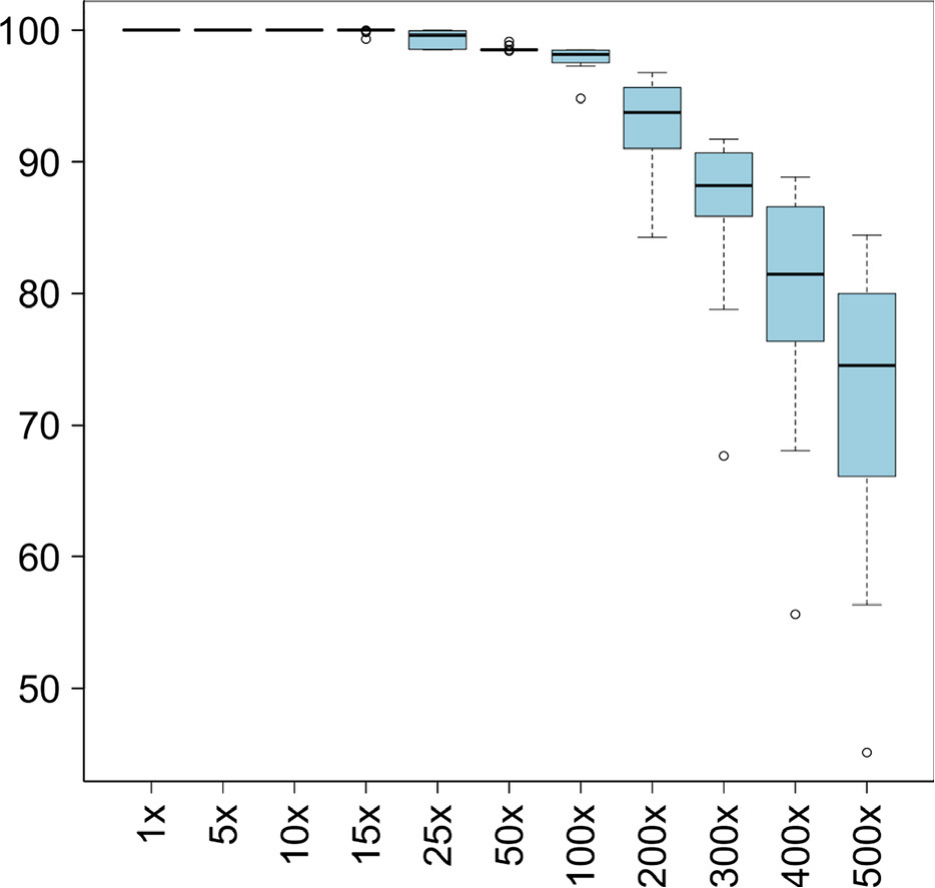
Percentage of target bases covered at different sequencing depths in 21 Finnish PAH patient samples. Results from the sequencing coverage analyses are shown as percentage of targeted bases with >1×, >5×, >10×, >15×, >25×, >50X, >100×, and until >500× depth. To generate highly confident genotype calls, >15× coverage is considered to be enough.

**Figure 3:**
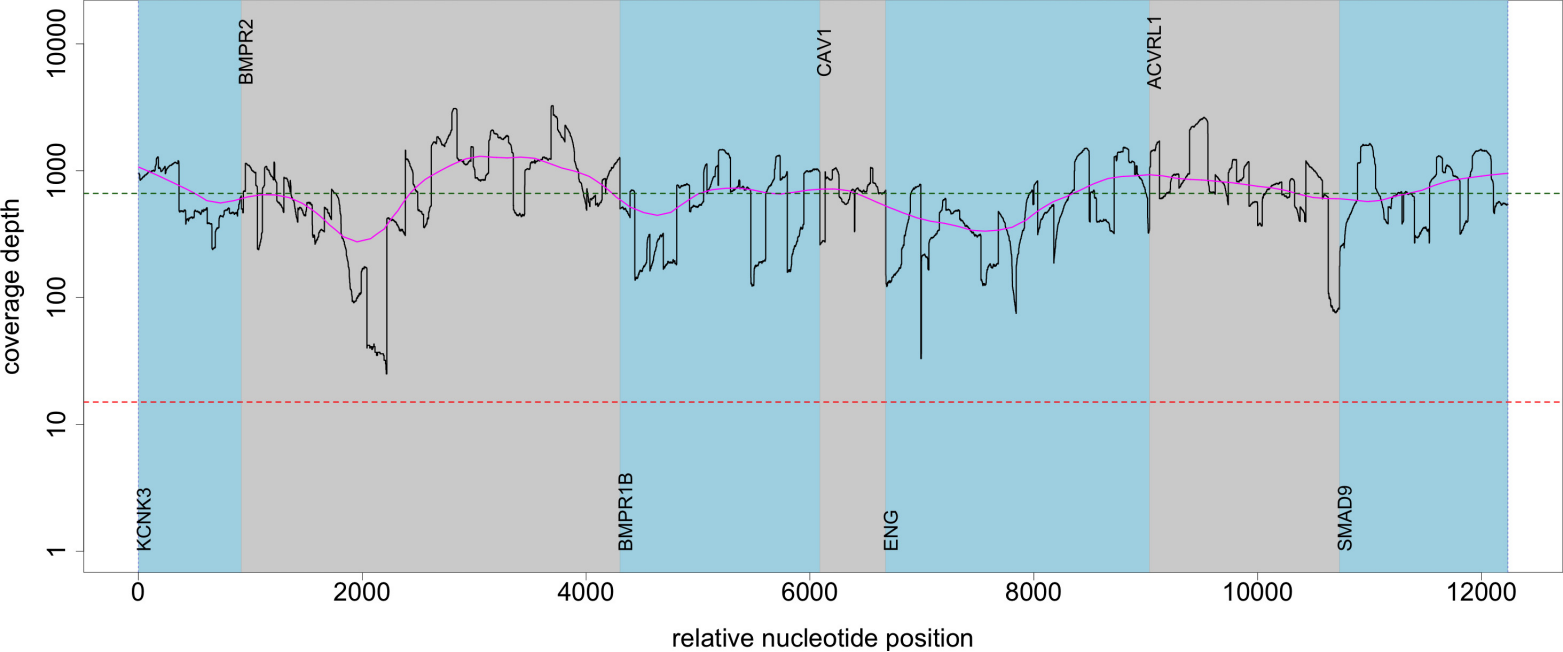
Sequencing coverage profile of an individual patient sample. The coverage profile of an individual patient shows how OS-Seq PAH panels with median sequencing depth of 662 and covered 100% target regions with >15× coverage. Coverage depth in *y*-axis shows the average of reads the certain nucleotide is sequenced. Relative nucleotide position is shown in *x*-axis.

**Figure 4:**
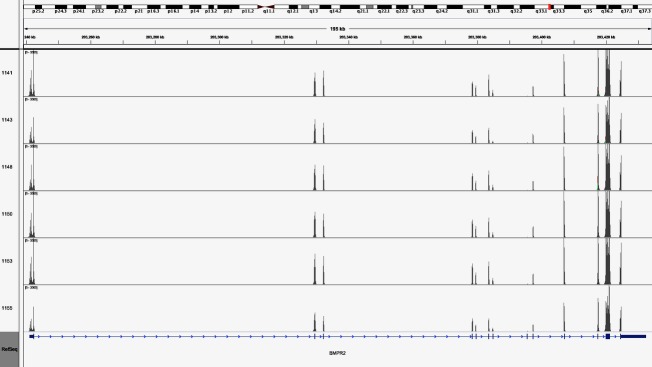
Coverage plot of coding exons of the *BMPR2* gene. The sequencing depth in the coding exons of the *BMPR2* gene in six PAH patient samples were sequenced in at high depths. The median coverage was 978 and coverage of nucleotides with >15× sequencing depth was 100%.

We evaluated the average sequencing depth of the PAH panel for the DNA samples of the 21 Finnish PAH patients by measuring the number of overlapping sequencing reads at each nucleotide position in the 12,638 base target region. The median sequencing depth was 791× and coverage of nucleotides with >15× sequencing depth was 100%. To demonstrate the sequencing coverage performance of the PAH panel we calculated the percentage of nucleotides exceeding specific sequencing depth threshold (1×–500×) (Fig.2). To demonstrate further the efficiency of targeted sequencing, the sequencing depth was also evaluated for individual patient samples (Fig.3) showing that 100% of the target regions were covered with >15× sequencing depth. Furthermore, we showed that the sequencing depth in the coding exons of the *BMPR2* gene in six PAH patient samples were sequenced in at high depth, as the median coverage was 978× and the coverage of nucleotides with >15× coverage was 100% (Fig.4).

## Discussion

Diagnostics of inherited diseases has entered a new era, in which sophisticated sequencing and bioinformatics reveal pathogenic mutations efficiently. There is estimated to be more than 7000 disorders with Mendelian inheritance and for many of these diseases the molecular genetic mechanism has been discovered. Although individual inherited diseases are rare, together they impose a significant clinical and economical challenge (Costa et al. 1985). The increasing realization of genetic underpinnings of inherited diseases has led to diagnostic challenges. Obtaining a complete genetic view of the disease requires high-quality sequencing of large number of genes and genomic regions with explicit clinical relevance and is beyond the scope and capacity of Sanger sequencing applications. At the same time, treatment of inherited diseases in the clinic presumes faster turnaround time and cost-effectiveness from genetic testing. Therefore, long odysseys of mutation exclusion by queuing Sanger sequencing experiments are already outdated in the clinic. Novel technologies have emerged to fulfill the growing clinical demand and to overcome the challenges associated with genetic testing of inherited diseases.

Targeted next-generation sequencing (NGS) is a practical solution for diagnostic-grade and comprehensive analysis of the clinically relevant genomic regions. While sequencing whole genomes or exomes are powerful tools for discovery and research, they fall short meeting the quality requirements of genetic diagnostics. Typical genome with 30× coverage or exome even with 100× coverage contains gaps and regions where sequence information remains unreliable (Meynert et al. 2013; Rehm et al. 2013; Sims et al. 2014). In order to reach consummate clinical genetic interpretation and solid diagnosis, those regions where data are narrow or missing need to be patched with alternative approaches, such as Sanger Sequencing. Necessity of gap-filling makes application of whole-genome sequencing (WGS) and whole-exome sequencing (WES) for genetic diagnostics uneconomical and impractical. Targeted NGS approaches enable cost-efficient, reliable, and high-depth sequencing of clinically relevant genomic regions with complete coverage.

To demonstrate the utility of NGS in the diagnostics of inherited diseases, we have developed an OS-Seq sequencing panel to accurately detect mutations in seven genes that have been implicated in the pathogenesis of PAH. We collected over 900 mutations from literature and existing databases to support the interpretation of the patients’ variants. Over 90% of the collected mutations were base substitutions causing missense, nonsense, and splice site mutations or small indels of up to 20 bp that can be identified with NGS (Fig.1).

The overall performance of our test was high. Sequencing depth and coverage are the main indicators of the performance of NGS. Sequencing depth refers to the number of sequencing reads that pileup at specific nucleotide and is directly associated with the confidence of the genotype call. The average (measured as median) sequencing depth was 791, which illustrates the confidence in calling the genotypes. Sequencing coverage defines the breadth of the genomic area, which has sequencing depth exceeding a specific threshold. Sequencing coverage is related to sensitivity of detecting variants in the target regions (Sims et al. 2014). We regarded target regions being covered when exceeding a 15× sequencing depth. It has been demonstrated that a sequencing depth of at least 15× is appropriate for making confident calls, as calling heterozygous variants rarely requires more than 13 overlapping sequence reads (Meynert et al. 2013; Sims et al. 2014). Sequencing coverage at >15× depth for our PAH panel was 100% indicating a high sensitivity to detect variants in the target regions.

We analyzed 21 Finnish PAH patients using our novel, targeted OS-Seq panel for PAH. Although the study cohort was small it was able to confirm relatively high incidence (29%) of pathogenic or likely pathogenic variants in *BMPR2* gene among Finnish PAH patients (Table2). In this cohort, the analysis of other PAH-associated genes did not reveal any other genetic variants considered significant (Table3), further pinpointing the pivotal role that *BMPR2* has in the pathogenesis of PAH. Importantly, with our OS-Seq approach, we identified all previously detected *BMPR2* mutations and identified two individuals with pathogenic or likely pathogenic *BMPR2* gene variant that were previously missed by Sanger sequencing. In the Sanger method, nonspecific binding of the primers and the formation of DNA secondary structures may cause sequencing errors (Hert et al. 2008; Sanger et al. 1977). There is also increased risk for human error when interpreting the raw sequencing results due to ambiguities in the capillary electrophoresis readouts. These factors could have caused the failures to identify the two *BMPR2* mutations in the primary study (Sankelo et al. 2005).

*ACVRL1* or *ENG* mutations are often found in PAH patients with family history of hereditary hemorrhagic telangiectasia (HHT) (Harrison et al. 2003). As none of the studied Finnish PAH patients manifested clear HHT-disease, it was not surprising that the analysis of *ACVRL1* and *ENG* did not identify any pathogenic variants (Table3). The patient with the variant in the *ACVRL1* gene (c.536A>C, p.Asp179Ala) also carried a pathogenic deletion in the *BMPR2* gene (c.1376_1377delGA, p.Arg459ThrfsX11) (Table2). Interestingly, this pathogenic *BMPR2* variant resulting a frameshift was missed in previous study (Sankelo et al. 2005) using Sanger sequencing and the *ACVRL1* mutation was considered a potential cause for PAH although the patient lacked clear symptoms associated with HHT. As the patient is carrying a *BMPR2* variant resulting a deletion and is considered to be pathogenic, we suggest that it is unlikely that this *ACVRL1* variant is an independent disease-causing mutation. The identified *ENG* gene variant, featuring a missense mutation (c.14C>T, p.Thr5Met; rs35400405), from two patients is interesting as it leads to the replacement of threonine by methionine resulting a suppression of *ENG* expression (Bourdeau et al. 2001). Despite its direct effects on protein (Bourdeau et al. 2001) it is predicted to be non-disease causing as the allele is common in general population (MAF 0.04790) (Table3).

Characterization of mutations in PAH enables establishing estimates on prognosis. *BMPR2* mutations are associated with an earlier age of disease onset, regardless of familial history, and with more severe disease phenotype. Furthermore, patients with *BMPR2* mutations are less likely to response to vasodilators than mutation-negative patients (Austin et al. 2009; Ma and Chung 2014). PAH patients carrying mutations in *ACVRL1* gene show more rapid disease progression than patients with *BMPR2* mutations, despite responding to vasodilators at the time of diagnosis (Girerd et al. 2010). In addition, patients identified with *BMPR2* and *ACVRL1* mutations have a shorter survival time or earlier need for lung transplantation (Austin et al. 2009; Chida et al. 2012b; Girerd et al. 2010; Ma and Chung 2014). The study of Austin and coworkers in 2009 reported that patients with missense mutations in *BMPR2* gene are diagnosed in much younger age and seem to have a significantly shorter survival time compared to patients with truncating mutations (Austin et al. 2009). In 2012, Liu with colleagues reported male *BMPR2* mutation carriers had a worse prognosis compared to female mutation carriers (Liu et al. 2012). Pediatric PAH patients, with or without *BMPR2* mutation, show distinct response to vasodilators than adults, and are more likely to associate with other genetic syndromes (Ma and Chung 2014).

Utilization of novel targeted OS-Seq approach increases the diagnostic efficacy by offering better quality and faster turnaround time. As shown in this study, OS-Seq technology proved to be a practical and effective tool for genetic profiling of Finnish patients with PAH. As genetic testing is increasingly applied in clinical practice, it is important to acknowledge the requirements of quality and performance of available genetic tests. Diagnostic-grade deep sequencing with 100% covered target base pairs is becoming a standard requirement in today’s clinical genetic testing. The high incidence of disease mutations in patients with IPAH and HPAH and elevated estimates of disease penetrance are supporting the utilization of genetic testing as routine procedure in the evaluation of patients with IPAH or HPAH. As in other hereditary cardiovascular diseases genetic diagnosis can significantly rationalize the risk stratification and follow-up strategies in the family and can have impact in estimating index patient’s prognosis. New sequencing strategies and bioinformatics tools are enabling diagnostics with faster turnaround times and decreased cost.

## References

[b1] Adzhubei IA, Schmidt S, Peshkin L, Ramensky VE, Gerasimova A, Bork P (2010). A method and server for predicting damaging missense mutations. Nat. Methods.

[b2] Austin ED, Phillips JA, Cogan JD, Hamid R, Yu C, Stanton KC (2009). Truncating and missense BMPR2 mutations differentially affect the severity of heritable pulmonary arterial hypertension. Respir. Res.

[b3] Austin ED, Ma L, LeDuc C, Berman Rosenzweig E, Borczuk A, Phillips JA (2012). Whole exome sequencing to identify a novel gene (caveolin-1) associated with human pulmonary arterial hypertension. Circ. Cardiovasc. Genet.

[b4] Bourdeau A, Faughnan ME, McDonald ML, Paterson AD, Wanless IR, Letarte M (2001). Potential role of modifier genes influencing transforming growth factor-beta1 levels in the development of vascular defects in endoglin heterozygous mice with hereditary hemorrhagic telangiectasia. Am. J. Pathol.

[b5] Chakinala MM (2005). Changing the prognosis of pulmonary arterial hypertension: impact of medical therapy. Semin. Respir. Crit. Care Med.

[b6] Chaouat A, Coulet F, Favre C, Simonneau G, Weitzenblum E, Soubrier F (2004). Endoglin germline mutation in a patient with hereditary haemorrhagic telangiectasia and dexfenfluramine associated pulmonary arterial hypertension. Thorax.

[b7] Chida A, Shintani M, Nakayama T, Furutani Y, Hayama E, Inai K (2012a). Missense mutations of the BMPR1B (ALK6) gene in childhood idiopathic pulmonary arterial hypertension. Circ. J.

[b8] Chida A, Shintani M, Yagi H, Fujiwara M, Kojima Y, Sato H (2012b). Outcomes of childhood pulmonary arterial hypertension in BMPR2 and ALK1 mutation carriers. Am. J. Cardiol.

[b9] Costa T, Scriver CR, Childs B (1985). The effect of Mendelian disease on human health: a measurement. Am. J. Med. Genet.

[b10] Girerd B, Montani D, Coulet F, Sztrymf B, Yaici A, Jais X (2010). Clinical outcomes of pulmonary arterial hypertension in patients carrying an ACVRL1 (ALK1) mutation. Am. J. Respir. Crit. Care Med.

[b11] Harrison RE, Flanagan JA, Sankelo M, Abdalla SA, Rowell J, Machado RD (2003). Molecular and functional analysis identifies ALK-1 as the predominant cause of pulmonary hypertension related to hereditary haemorrhagic telangiectasia. J. Med. Genet.

[b12] Hert DG, Fredlake CP, Barron AE (2008). Advantages and limitations of next-generation sequencing technologies: a comparison of electrophoresis and non-electrophoresis methods. Electrophoresis.

[b13] Larkin EK, Newman JH, Austin ED, Hemnes AR, Wheeler L, Robbins IM (2012). Longitudinal analysis casts doubt on the presence of genetic anticipation in heritable pulmonary arterial hypertension. Am. J. Respir. Crit. Care Med.

[b14] Li H, Durbin R (2009). Fast and accurate short read alignment with Burrows-Wheeler transform. Bioinformatics.

[b15] Li X, Zhang X, Leathers R, Makino A, Huang C, Parsa P (2009). Notch3 signaling promotes the development of pulmonary arterial hypertension. Nat. Med.

[b16] Liu D, Wu WH, Mao YM, Yuan P, Zhang R, Ju FL (2012). BMPR2 mutations influence phenotype more obviously in male patients with pulmonary arterial hypertension. Circ. Cardiovasc. Genet.

[b17] Ma L, Chung WK (2014). The genetic basis of pulmonary arterial hypertension. Hum. Genet.

[b18] Ma L, Roman-Campos D, Austin ED, Eyries M, Sampson KS, Soubrier F (2013). A novel channelopathy in pulmonary arterial hypertension. N. Engl. J. Med.

[b19] Machado RD, Pauciulo MW, Thomson JR, Lane KB, Morgan NV, Wheeler L (2001). BMPR2 haploin-sufficiency as the inherited molecular mechanism for primary pulmonary hypertension. Am. J. Hum. Genet.

[b20] Machado RD, Aldred MA, James V, Harrison RE, Patel B, Schwalbe EC (2006). Mutations of the TGF-beta type II receptor BMPR2 in pulmonary arterial hypertension. Hum. Mutat.

[b21] Machado RD, Eickelberg O, Elliott CG, Geraci MW, Hanaoka M, Loyd JE (2009). Genetics and genomics of pulmonary arterial hypertension. J. Am. Coll. Cardiol.

[b22] McKenna A, Hanna M, Banks E, Sivachenko A, Cibulskis K, Kernytsky A (2010). The genome analysis toolkit: a MapReduce framework for analyzing next-generation DNA sequencing data. Genome Res.

[b23] Meynert AM, Bicknell LS, Hurles ME, Jackson AP, Taylor MS (2013). Quantifying single nucleotide variant detection sensitivity in exome sequencing. BMC Bioinfor.

[b24] Myllykangas S, Buenrostro JD, Natsoulis G, Bell JM, Ji HP (2011). Efficient targeted resequencing of human germline and cancer genomes by oligonucleotide-selective sequencing. Nat. Biotechnol.

[b25] Ng PC, Henikoff S (2001). Predicting deleterious amino acid substitutions. Genome Res.

[b26] Ng PC, Henikoff S (2002). Accounting for human polymorphisms predicted to affect protein function. Genome Res.

[b27] Rehm HL, Bale SJ, Bayrak-Toydemir P, Berg JS, Brown KK, Deignan JL (2013). ACMG clinical laboratory standards for next-generation sequencing. Genet. Med.

[b28] Runo JR, Loyd JE (2003). Primary pulmonary hypertension. Lancet.

[b29] Sanger F, Nicklen S, Coulson AR (1977). DNA sequencing with chain-terminating inhibitors. Proc. Natl. Acad. Sci. USA.

[b30] Sankelo M, Flanagan JA, Machado R, Harrison R, Rudarakanchana N, Morrell N (2005). BMPR2 mutations have short lifetime expectancy in primary pulmonary hypertension. Hum. Mutat.

[b31] Schmieder R, Edwards R (2011). Quality control and preprocessing of metagenomic datasets. Bioinformatics.

[b32] Schwarz JM, Cooper DN, Schuelke M, Seelow D (2014). MutationTaster2: mutation prediction for the deep-sequencing age. Nat. Methods.

[b33] Shintani M, Yagi H, Nakayama T, Saji T, Matsuoka R (2009). A new nonsense mutation of SMAD8 associated with pulmonary arterial hypertension. J. Med. Genet.

[b34] Sims D, Sudbery I, Ilott NE, Heger A, Ponting CP (2014). Sequencing depth and coverage: key considerations in genomic analyses. Nat. Rev. Genet.

[b35] Soubrier F, Chung WK, Machado R, Grunig E, Aldred M, Geraci M (2013). Genetics and genomics of pulmonary arterial hypertension. J. Am. Coll. Cardiol.

